# Convalescent Plasma for the Treatment of Severe COVID‐19 Infection in Cancer Patients

**DOI:** 10.1002/cam4.3457

**Published:** 2020-09-17

**Authors:** Douglas Tremblay, Carina Seah, Thomas Schneider, Sheena Bhalla, Jonathan Feld, Leonard Naymagon, Bo Wang, Vaibhav Patel, Tomi Jun, Thomas Jandl, Farah Rahman, Sean T. H. Liu, Judith A. Aberg, Nicole Bouvier, The Mount Sinai Health System Convalescent Plasma Team

**Affiliations:** ^1^ Division of Hematology and Medical Oncology Icahn School of Medicine at Mount Sinai Tisch Cancer Institute New York NY USA; ^2^ Graduate School of Biomedical Science Icahn School of Medicine at Mount Sinai New York NY USA; ^3^ Department of Pathology Icahn School of Medicine at Mount Sinai New York NY USA; ^4^ Division of Infectious Diseases Department of Medicine Icahn School of Medicine at Mount Sinai New York NY USA

**Keywords:** cancer, convalescent plasma, COVID‐19, malignancy, SARS‐CoV‐2

## Abstract

**Background:**

Patients with malignancy are particularly vulnerable to infection with Severe Acute Respiratory Disease‐Coronavirus‐2 (SARS‐CoV‐2) given their immunodeficiency secondary to their underlying disease and cancer‐directed therapy. We report a case series of patients with cancer who received convalescent plasma, an investigational therapy for severe Coronavirus Disease 2019 (COVID‐19).

**Methods:**

Patients with cancer were identified who received convalescent plasma. Enrolled patients had confirmed COVID‐19 with severe or life‐threatening disease and were transfused with convalescent plasma from donors with a SARS‐CoV‐2 anti‐spike antibody titer of ≥ 1:320 dilution. Oxygen requirements and clinical outcomes of interests were captured as well as laboratory parameters at baseline and 3 days after treatment.

**Results:**

We identified 24 patients with cancer, 14 of whom had a hematological malignancy, who were treated with convalescent plasma. Fifteen patients (62.5%) were on cancer‐directed treatment at the time of COVID‐19 infection. After a median of hospital duration of 9 days, 13 patients (54.2%) had been discharged home, 1 patient (4.2%) was still hospitalized, and 10 patients had died (41.7%). Non‐intubated patients, particularly those on nasal cannula alone, had favorable outcomes. Three mild febrile non‐hemolytic transfusion reactions were observed. C‐reactive protein significantly decreased after 3 days of treatment, while other laboratory parameters including ferritin and D‐dimer remained unchanged.

**Conclusions:**

Convalescent plasma may be a promising therapy in cancer patients with COVID‐19.

## Graphical abstract

Patients with cancer are particularly vulnerable to infection with COVID‐19 given immunodeficiency secondary to their underlying disease and cancer‐directed therapy. Here, we report a case series of 24 cancer patients with COVID‐19 treated with convalescent plasma. Non‐intubated patients, particularly those on nasal cannula alone, had favorable outcomes, suggesting convalescent plasma may be a viable treatment options for cancer patients with COVID‐19, particularly if utilized early in the disease course.

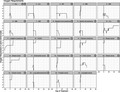

## 1 INTRODUCTION

Coronavirus disease 2019 (COVID‐19) is a global pandemic which disproportionally affects patients with cancer.^1^, ^2^ The scale of COVID‐19–related morbidity and mortality in cancer patients is unknown but likely varies geographically. In a meta‐analysis of early reports from China, the prevalence of malignancy in COVID‐19 patients was 0.92%^3^; however, in a large study based in New York City the prevalence was 5.6%.^4^ Importantly, the mortality rate in cancer patients with COVID‐19 is disproportionally high. In a report of 218 symptomatic COVID‐19 patients with cancer, 28% died, which was more than double that of age‐ and sex‐matched controls.^5^

Cancer patients are disproportionately affected by COVID‐19 for multiple reasons. Many patients are immunocompromised as a consequence of their underlying disease and/or cancer‐directed treatment, which often includes myelosuppressive chemotherapy, immunosuppressive targeted agents, and radiation therapy. In addition, patients with cancer frequent healthcare centers, which have been implicated in COVID‐19 transmission.^6^ Patients with hematologic malignancies, in particular, may have perturbations in myeloid and lymphoid maturation that leave them especially susceptible to the exuberant cytokine storm associated with severe disease.^5^, ^7^ In addition, impaired humoral immunity is common in malignancy, which can lead to ineffective defenses against viruses and infected cells.^8^

At present, few treatment options exist for patients with COVID‐19. Convalescent plasma has been employed in prior pandemics, with some evidence of efficacy in patients with H1N1 influenza and SARS.^9^, ^10^ The proposed mechanism of convalescent plasma is primarily through transfer of virus‐neutralizing antibodies against COVID‐19. Recent observational studies have demonstrated its safety and potential effectiveness.^11^, ^12^, ^13^, ^14^, ^15^ However, reports to date have not described outcomes for patients with underlying cancers specifically. Herein, we describe the outcomes of 24 cancer patients who received convalescent plasma as part of an expanded access protocol (NCT04338360).

## 2 METHODS

### 2.1 Patients

The Convalescent Plasma Program at Icahn School of Medicine at Mount Sinai initiated convalescent plasma transfusions on March 28, 2020, via the United States Food and Drug Administration (FDA) single‐patient emergency investigational new drug (eIND) pathway, announced on March 24, 2020. All patients in this case series, however, were treated between April 11 and May 10, 2020 under the FDA’s national Expanded Access Protocol (EAP), an FDA‐initiated, national, multicenter, open‐label program whose lead institution is the Mayo Clinic (https://www.uscovidplasma.org/ ). Expanded access permits the use of an investigational therapy when the primary purpose is to diagnose, monitor, or treat a patient's disease or condition, rather than to obtain the kind of information that is generally derived from clinical trials. Under current FDA regulations, patients with serious or immediately life‐threatening diseases who lack other therapeutic alternatives may be treated with investigational agents under several categories of expanded access, one of which includes protocols, such as the current convalescent plasma EAP, that are designed for widespread use in large patient populations (21 CFR 312.320).

Eligible patients were 18 years or older; had confirmed COVID‐19, as determined by a positive result on a reverse‐transcriptase–polymerase‐chain‐reaction (RT‐PCR) SARS‐CoV‐2 assay of a nasopharyngeal swab specimen; were hospitalized; and had severe or life‐threatening disease or were judged to be at risk for severe or life‐threatening disease. Severe disease was defined by one or more of the following: dyspnea; respiratory frequency greater than or equal to 30 breaths per minute; blood oxygen saturation of less than or equal to 93%; partial pressure of arterial oxygen to fraction of inspired oxygen ratio of less than 300; or lung infiltrates occupying greater than 50% of lung fields. Life‐threatening disease was defined by one or more of the following: respiratory failure; septic shock; multi‐organ dysfunction or failure. Each patient, or a legally authorized representative, provided informed consent prior to transfusion. The study protocol was approved and overseen by the Mayo Clinic Institutional Review Board (IRB) (#20‐003312) and endorsed by Icahn School of Medicine at Mount Sinai IRB (#20‐03393).

Patients reported in this case series had a documented cancer diagnosis prior to COVID‐19. Active cancer patients were defined as those currently receiving cancer‐directed therapy or those with radiographic or pathologic evidence of active disease. Patients were considered to be receiving cancer‐directed therapy if they had received treatment within the preceding 2 months prior to diagnosis of SARS‐CoV‐2 infection.

### 2.2 Convalescent plasma donation and preparation

Convalescent plasma donors were screened for SARS‐CoV‐2 antibody titers by a two‐step Spike protein‐directed ELISA.^16^ Donors with anti‐spike antibody titers ≥ 1:320 were referred for blood collection at the New York Blood Center, which performed the plasmapheresis and then returned convalescent plasma units to The Mount Sinai Hospital. Plasma recipients were transfused with two units of ABO‐compatible convalescent plasma. Each unit, approximately 250 milliliters in volume, was infused over 1 to 2 hours. Recipients were monitored every 15 minutes for signs of transfusion‐related reactions and then followed post‐transfusion for outcomes.

### 2.3 Data collection, definitions, and outcomes

Clinical information on all patients was obtained via the electronic medical record and included baseline demographic data, information related to their cancer diagnosis and treatment, and COVID‐19 treatment. Data were analyzed through June 22, 2020. Disease severity was quantified by a 7‐point ordinal scale used in previous COVID‐19 therapeutic trials and was developed by the World Health Organization (WHO) R&D Blueprint expert group^17^, ^18^: (a) not hospitalized with resumption of normal activities; (b) not hospitalized, but unable to resume normal activities; (c) hospitalized, not requiring supplemental oxygen; (d) hospitalized, requiring supplemental oxygen; (e) hospitalized, requiring nasal high‐flow oxygen therapy, noninvasive mechanical ventilation, or both; (f) hospitalized, requiring extracorporeal membrane oxygenation (ECMO), invasive mechanical ventilation, or both; and (g) death.

In addition to death, hospitalization status, and oxygenation requirements, we also assessed key safety information including transfusion reactions, which were defined and graded according to the Centers for Disease Control Hemovigilance Protocol.^19^ Finally, we examined the change in laboratory values of interest, including C‐reactive protein (CRP), ferritin, D‐dimer, and absolute lymphocyte count. These laboratory parameters were collected before convalescent plasma treatment and 3 days after, if the patient was alive and still hospitalized (these time periods were used in a prior convalescent plasma case series).^12^

### 2.4 Statistical analysis

Data presented in this case series are primarily descriptive. Laboratory values before and after convalescent plasma treatment were compared by Wilcoxon signed‐rank test. All data were assessed for normality using the Shapiro‐Wilk test with alpha = 0.05. Summary statistics for all data that were not normally distributed were reported as median ± interquartile range (IQR). Graphs were plotted using ggplot2_3.1.1 and ggpubr_0.2 R libraries. Statistical analysis was conducted in R using the dplyr_0.8.5 library.

## 3 RESULTS

### 3.1 Patient characteristics

We identified 24 cancer patients who were treated with convalescent plasma for COVID‐19. As shown in Table 1, the majority of patients (n = 14, 58.3%) had a hematologic malignancy, the most common of which was non‐Hodgkin lymphoma of the following subtypes: follicular lymphoma (n = 2), diffuse large B‐cell lymphoma (n = 1), mantle cell lymphoma (n = 1), and marginal zone lymphoma (n = 1). With the exception of one patient with Hodgkin lymphoma in remission, with no evidence of disease, all patients with hematological malignancies had active disease. Eleven patients (45.8%) were on cancer‐directed treatment at time of COVID‐19 infection, with a median time from last therapy to convalescent plasma of 44 days (range 0‐59 days). One patient with myelofibrosis (patient 3) was on ruxolitinib at the time of COVID‐19 infection. There were no patients treated with selinexor, ibrutinib, acalabrutinib, or zanubritinib.

**TABLE 1:** Baseline patient characteristics

Age at diagnosis, median (range)	69 (31‐88)
Gender, N (%)	
Female	10 (41.7)
Male	14 (58.3)
Race/Ethnicity, N (%)	
Hispanic	11 (41.8)
White	6 (25.0)
Asian	4 (16.7)
Black	3 (12.5)
Hematologic malignancy, N (%)	14 (58.3)
Non‐Hodgkin lymphoma	5 (20.8)
Multiple myeloma	4 (16.7)
Acute lymphoblastic leukemia	2 (8.3)
Hodgkin lymphoma	1 (4.2)
Myelofibrosis	1 (4.2)
Chronic lymphocytic leukemia	1 (4.2)
Solid malignancy, N (%)	10 (41.7)
Colorectal	2 (8.3)
Breast	2 (8.3)
Endometrial	2 (8.3)
Prostate	1 (4.2)
Lung	1 (4.2)
Ovarian	1 (4.2)
Laryngeal	1 (4.2)
Stage of solid tumor, N (%)	
I	3 (30.0)
II	3 (30.0)
III	4 (40.0)
Active disease, N (%)	17 (70.8)
Receiving cancer‐directed treatment at time of convalescent plasma, N (%)	11 (45.8)
Targeted therapy	9 (37.5)
Systemic chemotherapy	6 (25.0)
Radiation therapy	1 (4.2)
Immunomodulator	2 (8.3)
Intrathecal chemotherapy	1 (4.2)
Time from cancer diagnosis to convalescent plasma in months, median (range)	42.1 (2.3‐274.9)

Ten patients (41.7%) had a solid tumor, with breast, colorectal, and endometrial cancers being the most common. Four of these patients (40%) had active disease, and these same patients were receiving active therapy at time of COVID‐19 infection. The median time from last therapy to convalescent plasma was 35 days (range 12‐91 days) None of the patients in this series had metastatic disease, although this was not an exclusion criterion for convalescent plasma treatment. One patient had staging imaging performed at an outside institution, and therefore staging was unknown.

Other comorbidities present in the cohort include hypertension in 15 patients (62.5%), diabetes in eight patients (33.3%), chronic kidney disease in seven patients (29.2%), coronary artery disease in five patients (20.8%), chronic obstructive pulmonary disease in five patients (20.8%), and congestive heart failure in two patients (8.3%). In terms of smoking status, four patients (16.7%) were current smokers and 10 patients (41.7%) were former smokers. Five patients (20.8%) were obese and 11 patients (45.8%) were overweight. At time of convalescent plasma transfusion, no patients were neutropenic (absolute neutrophil count less than 1.0 × 10^9^ cells/L), however, 14 patients (58.3%) were lymphocytopenic (absolute lymphocyte count less than 1.0 × 10^9^_cell_s/L).

Table 2 details prior COVID‐19 treatments and baseline oxygen status. Prior COVID‐19 specific therapies include combination of hydroxychloroquine and azithromycin in 13 patients (54.2%), hydroxychloroquine alone in three patients (12.5%), and azithromycin alone in two patients (8.3%), and remdesivir in two patients (8.3%). One patient (4.2%) received tocilizumab prior to convalescent plasma infusion. Sixteen patients (66.7%) received hydroxycholoroquine and 15 patients (62.5%) received azithromycin; at the time of treatment, limited evidence suggested that these drugs might be beneficial in COVID‐19, which has since been reconsidered.^20^, ^21^ At time of treatment, all but one patient required supplemental oxygen. Four patients (16.7%) required non‐invasive positive pressure ventilation (NIPPV) or high‐flow nasal cannula (HFNC) and three patients (12.5%) were intubated on mechanical ventilation at time of convalescent plasma infusion.

**TABLE 2:** COVID‐19 characteristics at study entry

Prior hydroxychloroquine, N (%)	16 (66.7)
Prior azithromycin, N (%)	15 (62.5)
Prior remdesivir, N (%)	2 (8.3)
Prior tocilizumab, N (%)	1 (4.2)
Oxygen requirement, N (%)	
Room air	1 (4.2)
Nasal cannula	12 (50)
Tracheostomy collar	1 (4.2)
Non‐rebreather mask	3 (12.5)
HFNC	2 (8.3)
BIPAP	2 (8.3)
Mechanical ventilation	3 (12.5)

Abbreviations: BIPAP, bilevel positive airway pressure; HFNC, high flow nasal cannula.

### 3.2 Outcomes and safety

**FIGURE 1:**
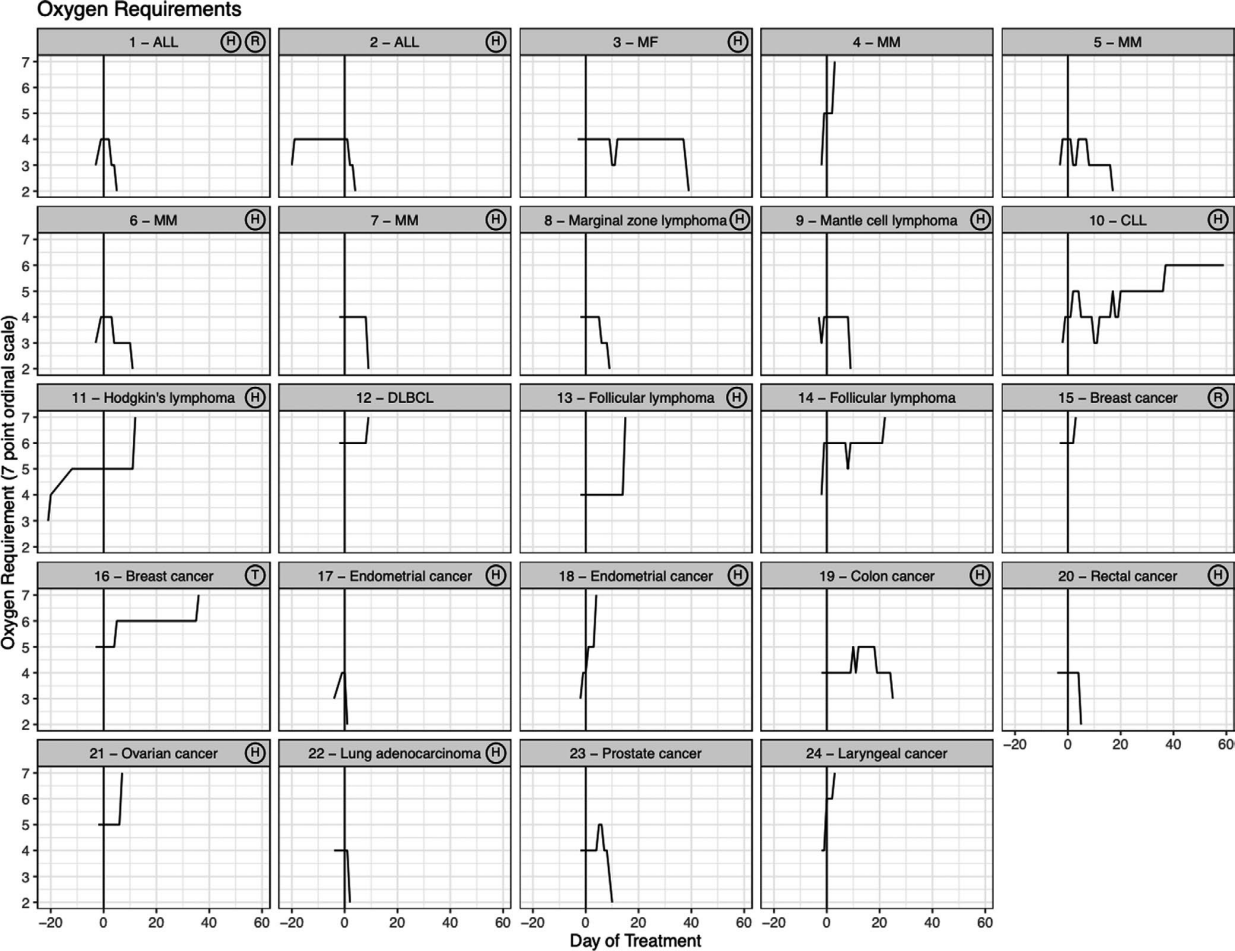
Temporal changes in oxygen requirement (7‐point ordinal scale) in patients treated with convalescent plasma. Each box represents the trajectory of oxygen requirement, measured by a 7‐point ordinal scale, over time for each individual patient. Noted in the upper right corner of each box is any additional COVID‐19 directed treatment the patient received. There was variability in clinical course after treatment with convalescent plasma. ALL, acute lymphoblastic leukemia; CLL, chronic lymphocytic leukemia; DLBCL, diffuse large B‐cell lymphoma; H, hydroxychloroquine; MF, myelofibrosis; MM, multiple myeloma; R, remdesivir; T, tocilizumab

Patients had a median hospital duration of 9 days (IQR 4‐15.5 days). The median time from hospital admission to convalescent plasma treatment was 3 days (IQR 2‐7 days). Thirteen patients (54.2%) were discharged home. As of June 22, 2020, one patient was still hospitalized. Ten patients died (41.7%). Nine patients died of progressive respiratory failure and one (patient 16) expired after a subarachnoid hemorrhage while on ECMO. Figure 1 shows the trajectory of oxygen requirements over time for all 24 patients, measured by the 7‐point ordinal scale. There was marked variability in both the timing and degree of improvement or worsening of oxygen requirement, with some patients experiencing rapid improvement (patients 1, 2, 6). Two patients were discharged soon after convalescent plasma infusion (patients 17, 22). Other patients had more gradual improvements (patients 6, 7, 8, 9, 23) or worsened (patients 11, 12, 13, 14). Finally, some patients had rapid decompensation (patients 4, 15,18, 24). Notably, all three patients who were intubated at time of convalescent plasma infusion (patients 12, 14, 15) expired. Three of the four patients on NIPPV or HFNC (ordinal oxygen scale of 5) at time of convalescent plasma enrollment expired. In contrast, only three patients (17.6%) on supplemental oxygenation via nasal cannula or tracheostomy collar expired.

Three patients experienced a transfusion reaction, all of which were febrile non‐hemolytic transfusion reactions (FNHTR). These were all determined to be non‐severe and imputability was determined to be probable. No other transfusion reactions were noted.

### 3.3 Change in laboratory parameters

**FIGURE 2:**
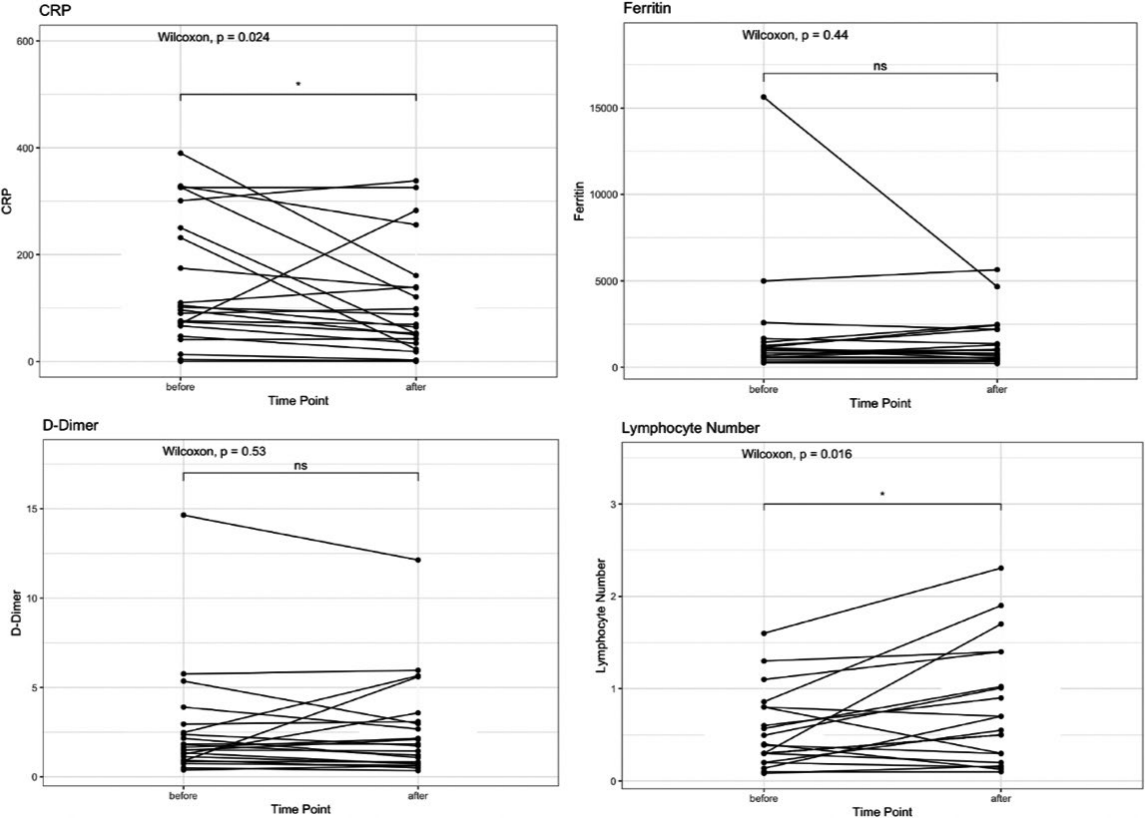
Pre‐ and Post‐transfusion changes in laboratory parameters for patients treated with convalescent plasma. Laboratory values were measured at baseline and 3 d after convalescent plasma infusion, if available. There was a significant decrease in C‐reactive protein after convalescent plasma infusion (*P* = .024) and a significant increase in lymphocyte count (*P* = .016), while D‐dimer and ferritin had no significant changes

We compared C‐reactive protein (CRP), ferritin, D‐dimer, and lymphocyte percentage before and 3 days after convalescent plasma infusion. As shown in Figure 2, there were significant decreases in CRP, with 13 patients (59.1%) experiencing improvement. In addition, there were significant increases in absolute lymphocyte numbers, with 15 patients (71.4%) having an increase from baseline to day 3. Of note, the one patient with chronic lymphocytic leukemia (CLL) was excluded from the lymphocyte count analysis, because the lymphocyte response to therapy could not be characterized, given the underlying malignancy. There was no significant change in ferritin and D‐dimer before and after convalescent plasma infusion in this small case series.

## 4 DISCUSSION

Patients with cancer are a particularly vulnerable population during the COVID‐19 pandemic and there remains a paucity of effective treatments.^22^ Convalescent plasma represents a compelling treatment option. However, outcomes limited to patients with cancer have not been reported. In this case series, we demonstrate that convalescent plasma appears safe and may help improve oxygen requirements in specific patients. Furthermore, there may be an anti‐inflammatory effect, as evidenced by a significant decrease in CRP, in select patients.

In our series, patients who received convalescent plasma while on mechanical ventilation, NIPPV, or HFNC had dismal survival. While this cohort is too small to draw definitive conclusions, this finding accords with prior studies^23^, ^24^ suggesting that convalescent plasma therapy may be most effective early in the course of the disease, prior to respiratory collapse, and may not be effective as a rescue agent. Given that the proposed mechanism of action of convalescent plasma is via neutralizing antibody transference to help viral clearance,^25^ this finding is not unexpected. Additionally, in patients with severe disease, an overexuberant inflammatory response is a key mediator of respiratory compromise.^7^ Convalescent plasma can theoretically provide anti‐inflammatory effects^25^; however, the clinical evidence for this is lacking. This suggests that convalescent plasma is more appropriate early in the disease course is and echoed by a separate cohort of patients from our institution^26^ and an underpowered randomized trial from China.^14^

Transfusion reactions were uncommon and mild, with only three patients experiencing a FNHTR. We did not observe other transfusion reactions, in particular transfusion‐related acute lung injury (TRALI) or transfusion associated circulatory overload (TACO), which could be particularly disastrous in patients with respiratory decompensation from COVID‐19. Another concern, albeit theoretical, is that convalescent plasma may promote thrombosis formation as it contains coagulation factors. This concern is compounded by emerging data on the procoagulant state of COVID‐19 and known thrombotic risk of malignancy.^27^, ^28^ Of note, there were no imaging confirmed thrombotic events in this case series after or before convalescent plasma infusion.

Patients with hematologic malignancies may be particularly vulnerable to COVID‐19, and preliminary evidence suggests that they have increased mortality as compared with the general population.^29^ In our series, all but one such patient was on active treatment, which has been associated with a more aggressive COVID‐19 course.^30^ Of the 14 patients with hematologic malignancies in our series, eight patients (57.1%) were discharged, one (7.1%) was still hospitalized and mechanically ventilated, and 5 (35.7%) expired. This high mortality rate likely reflects the severity of COVID‐19 infection in patients with hematologic malignancies. Notably, one patient with myelofibrosis (patients 3) was on ruxolitinib at the time of infection, which is currently being clinically investigated to combat hyperinflammation associated with COVID‐19.^31^ This patient never required intubation and it is possible that his clinical improvement may have been related to continuation of ruxolitinib during his hospitalization rather than convalescent plasma. There were no patients on Bruton Tyrosine Kinase inhibitors, selinixor, or other anti‐cancer therapies in active clinical trials for the treatment of COVID‐19.^32^

Two patients received investigational remdesivir prior to convalescent plasma infusion. The United States FDA has provided emergency use authorization with some reports suggesting a decreased time to recovery with this agent.^33^ Only one patient received tocilizumab, which observational studies suggest may be associated with a decreased rate of mechanical ventilation or death.^34^ Published randomized trials are not currently available for this agent, however, a press release reported that a phase III trial of tocilizumab failed to meets its primary endpoint of improved clinical status or key secondary endpoint of mortality.^35^

This case series has a number of limitations to consider. Aside from a limited sample size, there is an absence of a control cohort to reach any conclusive benefits with convalescent plasma treatment. It is worth noting that while donor anti‐SARS‐CoV‐2 spike antibodies titers were elevated (≥1:320), we have not yet assessed if these antibodies are neutralizing. In our study, there was also inconsistent availability and follow up on laboratory values, making it difficult to estimate the impact of convalescent plasma on relevant laboratory parameters. Additionally, there are a number of important confounders that can contribute to changes in laboratory values, in particularly concomitant therapies and interventions.

Despite these limitations, this series provides the clinical experience of convalescent plasma unique to patients with COVID‐19 who have an underlying malignancy. Although descriptive, this series supports further consideration of convalescent plasma as a therapeutic modality in patients with cancer, particularly when utilized early in the disease course prior to respiratory collapse.

## CONFLICT OF INTEREST

Judith A. Aberg receives grants and personal fees from Gilead, grants and personal fees from Merck, grants and personal fees from Janssen, personal fees from Theratech, personal fees from Medicure, grants from Regeneron, grants and personal fees from Viiv, outside of the submitted work. Sean T. H. Liu and Farah Rahman are sub‐investigators for Gilead, Regeneron, and Kinevan. These relationships are outside of the submitted work. The remaining authors have no conflicts of interest to disclose.

## AUTHOR CONTRIBUTIONS

Conceptualization and design: Douglas Tremblay, Carina Seah, Sean T. H. Liu, Judith A. Aberg, Nicole Bouvier. Data collection and curation: Douglas Tremblay, Carina Seah, Thomas Schneider. Patient recruitment: Douglas Tremblay, Sheena Bhalla, Jonathan Feld, Leonard Naymagon, Bo Wang, Vaibhav Patel, Tomi Jun, Thomas Jandl, Farah Rahman, Sean T. H. Liu, Nicole Bouvier. Drafting of article: Douglas Tremblay, Carina Seah, Sean T. H. Liu, Judith Aberg, Nicole Bouvier. All authors reviewed the manuscript. Douglas Tremblay and Carina Seah had full access to the data and take responsibility for the integrity of the data and the accuracy of the data analysis.

## Data Availability

The data that support the findings of this study are available from the corresponding author upon reasonable request and upon agreement from all coauthors.
